# CAB39L elicited an anti-Warburg effect via a LKB1-AMPK-PGC1α axis to inhibit gastric tumorigenesis

**DOI:** 10.1038/s41388-018-0402-1

**Published:** 2018-07-27

**Authors:** Weilin Li, Chi Chun Wong, Xiaoming Zhang, Wei Kang, Geicho Nakatsu, Qinfu Zhao, Huarong Chen, Minnie Yin Yin Go, Philip Wai Yan Chiu, Xiaohong Wang, Jiafu Ji, Xiaona Li, Zongwei Cai, Enders Kwok Wai Ng, Jun Yu

**Affiliations:** 1 Institute of Digestive Disease and Department of Medicine and Therapeutics, State Key Laboratory of Digestive Disease, Li Ka Shing Institute of Health Sciences, CUHK-Shenzhen Research Institute, The Chinese University of Hong Kong, Hong Kong, China; 2 Department of Surgery, The Chinese University of Hong Kong, Hong Kong, China; 3 Department of Anatomical and Cellular Pathology, The Chinese University of Hong Kong, Hong Kong, China; 4 Key laboratory of Carcinogenesis and Translational Research (Ministry of Education), Department of Gastrointestinal Surgery, Peking University Cancer Hospital and Institute, Beijing, China; 5 State Key Laboratory of Environmental and Biological Analysis, Department of Chemistry, Hong Kong Baptist University, Hong Kong, China

## Abstract

Metabolic dysfunction is a hallmark of gastric cancer (GC). In this study, we reported the identification of Calcium Binding Protein 39-Like (CAB39L) as a novel regulator of tumor metabolism in GC. CAB39L mRNA was frequently silenced by promoter methylation in GC cell lines and tissues. Functional studies suggested that CAB39L functions as a tumor suppressor, as overexpression of CAB39L elicited suppression of multiple cancer phenotypes both in GC cells and an orthotopic mouse model; whilst its knockdown promoted tumorigenesis. Mechanistically, CAB39L interacted with LKB1-STRAD complex and induced LKB1, leading to the phosphorylation and activation of AMPKα/β. LKB1-AMPK activation in GC cell lines was tumor suppressive, as metformin (an AMPK activator) inhibited GC cell growth in the CAB39L-silenced cells. Moreover, knockdown of LKB1 reversed growth inhibitory effect of CAB39L, indicating that tumor suppression by CAB39L depended on LKB1-AMPK. RNAseq and gene set enrichment analysis revealed that CAB39L was closely correlated with oxidative phosphorylation and mitochondrial biogenesis. Consistently, CAB39L-induced p-AMPK elicited PGC1α phosphorylation and increased the expression of genes involved in mitochondrial respiration complexes. Accordingly, CAB39L reversed the Warburg effect in GC, as evidenced by enhanced oxygen consumption rate and reduced extracellular acidification rate; inversely, CAB39L knockdown promoted a metabolic shift towards the Warburg phenotype. In GC patients, CAB39L promoter hypermethylation was correlated with poor prognosis. Our data demonstrate that CAB39L is a novel tumor suppressor which suppresses tumorigenesis by promoting LKB1-AMPK-PGC1α axis, thereby preventing a metabolic shift that drives carcinogenesis. CAB39L methylation is a potential prognostic biomarker for GC patients.

## Introduction

Gastric cancer (GC) continues to be a major cancer worldwide and GC is the second leading cause of cancer-related deaths [1, 2]. Despite tremendous efforts undertaken to improve GC treatment, including the implementation of preoperative neoadjuvant chemotherapy and postoperative chemo-radiotherapy [3, 4], GC remains to be a disease refractory to most therapeutic regimes with dismal prognosis, as most patients have inoperable disease at diagnosis or recurrence disease after resection [2, 5]. Hence, investigation into the molecular mechanisms underlying GC initiation and progression is significant.

Epigenetic dysregulation plays an important role in GC development [6]. In particular, DNA promoter hypermethylation has been shown to mediate transcriptional silencing of tumor suppressor genes in GC [7–11]. It is increasingly appreciated that epigenetic dysregulation acts in concert with cell metabolism to promote tumorigenesis [12, 13]. Metabolism of tumor cells differs significantly from that of normal cells. The Warburg Effect, first proposed by Otto Warburg, postulates that cancer suppresses oxidative phosphorylation and utilizes aerobic glycolysis for the generation of ATP [14, 15]. This shift in cellular metabolism is thought to promote tumorigenesis via rapid ATP turnover, increased biosynthesis and redox homeostasis in order to meet the requirement of uncontrolled cell growth regardless of oxygen or nutrient levels [15]. A bidirectional interaction exists between epigenetic and metabolomic dysregulation in cancers [12]. Numerous tumor-related metabolites have been shown to modify the epigenetic landscape [16–18]. On the other hand, the role of aberrant DNA methylation on tumor metabolism remains poorly understood [19, 20]. Nevertheless, few studies have assessed whether promoter hypermethylation contributes to metabolic rewiring in GC.

To uncover novel tumor suppressor genes that are epigenetically inactivated in GC, we performed Infinium Human Methylation 450 BeadChip to compare differentially methylated regions between four gastric cancer cell lines (AGS, HGC27, MGC803, and MKN45), and one normal gastric cell line (GES1) and gastric tissue samples. Based on our dataset, we have identified CAB39L as a novel gene hypermethylated in GC. CAB39L is located on chromosome 13q14.2 and is the β isoform of CAB39. CAB39L has been reported to be involved in reproductive cycle [21, 22]. However, the implication of CAB39L in GC development is largely unknown. In this work, we conducted the first study of CAB39L in GC. We revealed that CAB39L possesses tumor suppressive effects in GC cells, that CAB39L mediates its effect by eliciting an anti-Warburg effect via a LKB1-AMPK-PGC1α axis and that promoter methylation of CAB39L predicts poor outcomes in GC patients. CAB39L thus represents a novel metabolic checkpoint linking epigenetic dysregulation and metabolic rewiring in GC.

## Results

### CAB39L is silenced by promoter hypermethylation in gastric cancer cells

**Fig. 1:**
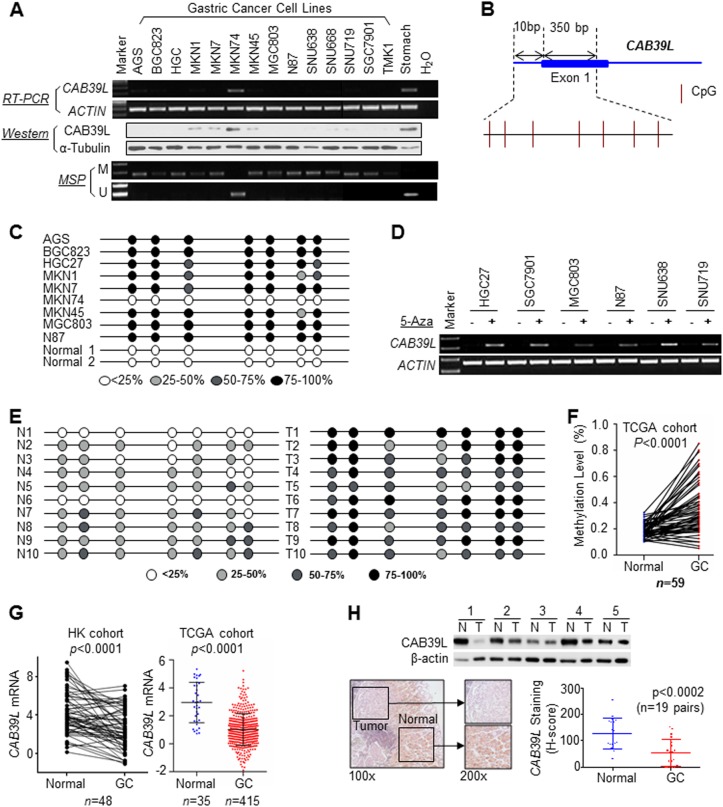
CAB39L was transcriptionally silenced via promoter methylation in gastric cancer. **a** CAB39L mRNA and protein level were down-regulated in 13 out of 14 gastric cancer cell lines, but was highly expressed in MKN74 and normal stomach tissue. Methylation-specific PCR (MSP) revealed CAB39L promoter hypermethylation in cell lines with silenced CAB39L. **b** Promoter region of CAB39L. CpG sites indicated for bisulfite genomic sequencing (BGS) were shown. **c** Promoter methylation in gastric cancer cell lines and normal gastric tissues were determined by BGS. **d** Demethylation treatment (5-Aza) restored CAB39L mRNA expression in gastric cancer cell lines. **e** BGS of CAB39L promoter revealed dense methylation in gastric cancer tissues as compared to adjacent normal tissues in Hong Kong cohort. **f** TCGA gastric cancer cohort confirmed promoter hypermethylation of CAB39L in gastric cancer (450 K methylation array) (*P* < 0.0001). **g** CAB39L mRNA expression was down-regulated in gastric cancer as compared to adjacent normal tissues in Hong Kong and the Cancer Genome Atlas (TCGA) cohorts (*P* < 0.0001). **h** CAB39L protein was down-regulated in gastric cancer as compared to adjacent normal tissues, as determined by Western blot and immunohistochemistry (lower, *P* < 0.0002)

Using Infinium Human Methylation 450 K Beadchip, we identified CpG sites in the promoter region of CAB39L were differentially methylated by over 45% (Δ*β*-value = 0.46) in GC cell lines compared to GES1 cells and adjacent normal gastric tissues (Figure S1). Integrative analyses of paired GC and normal gastric tissues from the Cancer Genomic Atlas (TCGA) cohort revealed that CAB39L was a top outlier gene with simultaneous DNA hypermethylation and decreased mRNA expression **(**Figure S1**)**. We performed methylation-specific PCR (MSP), RT-PCR and western blot to determine the promoter methylation status, mRNA and protein expression of CAB39L in 14 GC cell lines. As shown in Fig. 1a, MSP analysis indicated that 13 out of 14 (92.9%) GC cell lines showed promoter hypermethylation, except for MKN74 cells. mRNA expression analysis revealed that 13 out of 14 GC cell lines had low CAB39L mRNA expression; whilst exceptionally high CAB39L mRNA and protein expression were found in MKN74 cells and normal stomach tissue, consistent with the hypothesis that promoter hypermethylation mediates the transcriptional silence of CAB39L in GC. To validate CAB39L promoter methylation in GC cells, we performed bisulfite genomic sequencing (BGS) analysis of CpG sites in the CAB39L promoter and first exon (Fig. 1b). As expected, dense methylation (average CpG methylation > 50%) was found in 8 out of 9 GC cell lines; while being largely unmethylated in MKN74 cells and normal gastric tissues (Fig. 1c). To verify whether the silencing of CAB39L is due to the promoter methylation, 6 GC cell lines with CAB39L silenced were treated with 5-Aza-2′-deoxycytidine (5-Aza), a DNA methyltransferase inhibitor. In all cell lines, CAB39L expression was restored upon 5-Aza treatment (Fig. 1d). Collectively, our data indicated that silencing of CAB39L in GC cell lines is a consequence of promoter hypermethylation.

### CAB39L promoter hypermethylation contributes to its silencing in GC patients

Promoter methylation status was next examined in a cohort of 10 paired GC tumors and adjacent normal tissues by BGS (Fig. 1e), which revealed hypermethylation of CAB39L promoter in GC tumors. In an independent GC patient cohort from TCGA database (*n* = 59, paired), GC also exhibited promoter hypermethylation compared to adjacent normal tissues (*P* < 0.0001) (Fig. 1f). Next, we analyzed CAB39L mRNA expression in GC and adjacent normal tissues. In both HK (*n* = 48, paired, *P* < 0.0001) and TCGA GC cohort (*n* = 450, unpaired, *P* < 0.0001), CAB39L mRNA expression was significantly lower in GC tissues than that of adjacent normal tissues (Fig. 1g). Analysis of TCGA dataset revealed an inverse correlation between *CAB39L* promoter methylation and mRNA expression in GC (*P* < 0.001) (Figure S2). Finally, we evaluated protein expression of CAB39L in GC tissues. Western blot (*n* = 5, paired) and immunohistochemistry (*n* = 19, paired) confirmed the down-regulation of CAB39L protein in GC (Fig. 1h). These data suggested that CAB39L is silenced in human GC via promoter methylation.

### CAB39L suppresses GC growth by inducing apoptosis and cell cycle arrest

**Fig. 2:**
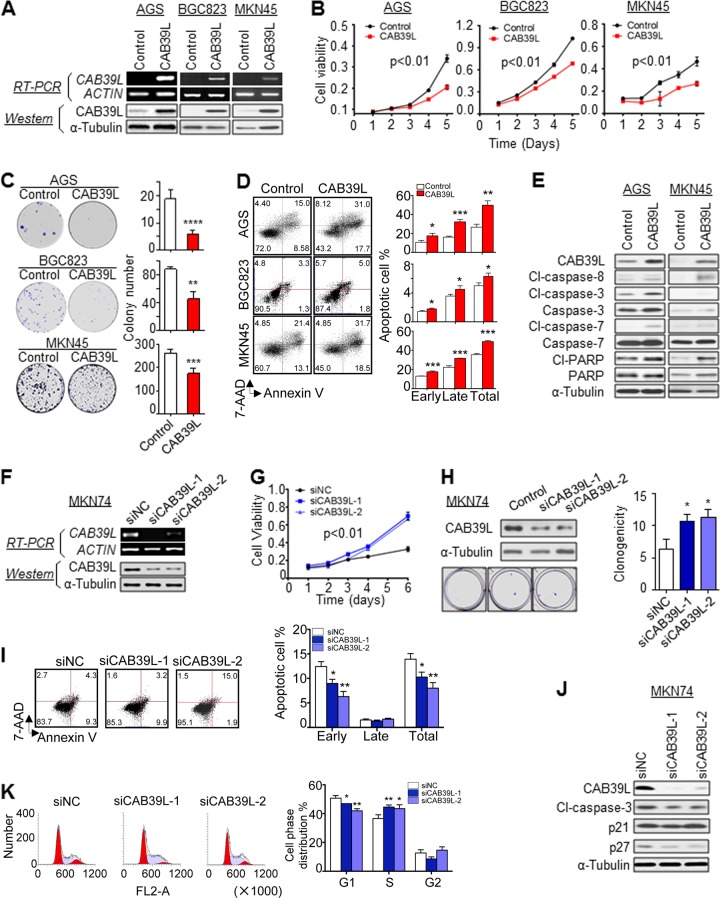
CAB39L functioned as a tumor suppressor in gastric cancer cell lines. **a** CAB39L was ectopically expressed in AGS, BGC823 and MKN45 cell lines. **b**, **c** CAB39L expression suppressed cell viability (*P* < 0.01) and colony formation ability in AGS (*P* < 0.0001), BGC823 (*P* < 0.01), and MKN45 (*P* < 0.001) cell lines. **d** CAB39L induced early and late stage apoptosis in AGS (*P* < 0.01), BGC823 (*P* < 0.05) and MKN45 cells (*P* < 0.001), as determined by Annexin V-PE/7-AAD staining and flow cytometry. **e** Western blot revealed that ectopic CAB39L expression induced the cleavage of caspase-3, -7, -8, and PARP, consistent with the induction of apoptosis. **f** CAB39L knockdown in MKN74 cells. **g**, **h** Knockdown of CAB39L promoted cell viability (*P* < 0.01) and colony formation ability (*P* < 0.05) in MKN74 cells. **i** CAB39L knockdown inhibited induction of early apoptosis (siCAB39L-1, *P* < 0.05; siCAB39L-2, *P* < 0.01). **j** Western blot demonstrated decreased expression of cleaved caspase-3, p21, and p27. **k** Knockdown of CAB39L in MKN74 cells promoted cell cycle progression from G1 to S phase (siCAB39L-1, *P* < 0.01; siCAB39L-2, *P* < 0.05). (*P < 0.05; **P < 0.01, ***P < 0.001, ****P < 0.001)

The frequent silencing of CAB39L in GC prompted us to hypothesize that CAB39L may function as a tumor suppressor. Therefore, we established three GC cell lines (AGS, BGC823 and MKN45) stably overexpressing CAB39L. Ectopic expression of CAB39L was validated by RT-PCR and Western blot (Fig. 2a). Cell growth curve indicated that CAB39L inhibited cell viability in all three cell lines (Fig. 2b, *P* < 0.01). We also tested cell viability in physiologically-relevant culture condition (5 mmol/L D-glucose, 0.8 mmol/L L-glutamine), and showed that CAB39L also maintained its tumor-suppressive effect (Figure S3A). In accordance with MTT assay, colony formation assay also showed reduced cell proliferation in the form of decreased size and number of colonies (Fig. 2c, AGS: *P* < 0.0001, MKN45: *P* < 0.001, BGC823: *P* < 0.01). To determine the cytokinetic effect of CAB39L on GC cells, we next evaluated apoptosis and cell cycle by flow cytometry. Overexpression of CAB39L in AGS, BGC823 and MKN45 cells resulted in increased apoptosis (both early and late phases) compared with the control cells (Fig. 2d). Consistent with induction of apoptosis, western blot showed increased expression of cleaved caspase-3, -7, -8, and PARP (Fig. 2e). Concomitantly, CAB39L silencing using two independent siRNAs in MKN74 cells (Fig. 2f) increased cell viability (Fig. 2g, *P* < 0.01) and colony formation (Fig. 2h, *P* < 0.05), consistent with the function of CAB39L as a tumor suppressor. Besides, rescue experiment by re-expression of CAB39L in MKN74- shCAB39L cells restored the tumor-suppressive effect of CAB39L (Figure S3B). Moreover, CAB39L knockdown in MKN74 cells suppressed induction of apoptosis (Fig. 2i, *P* < 0.05) and promoted cell cycle progression from G_1_ to S phase (Fig. 2k, *P* < 0.05). Western blot showed that CAB39L knockdown resulted in decreased expression of cleaved caspase-3, p21 and p27 (Fig. 2j). Collectively, these data suggest CAB39L exerts a tumor suppressive effect on GC by inducing apoptosis and cell cycle arrest.

### CAB39L suppresses GC cell invasion and migration

**Fig. 3:**
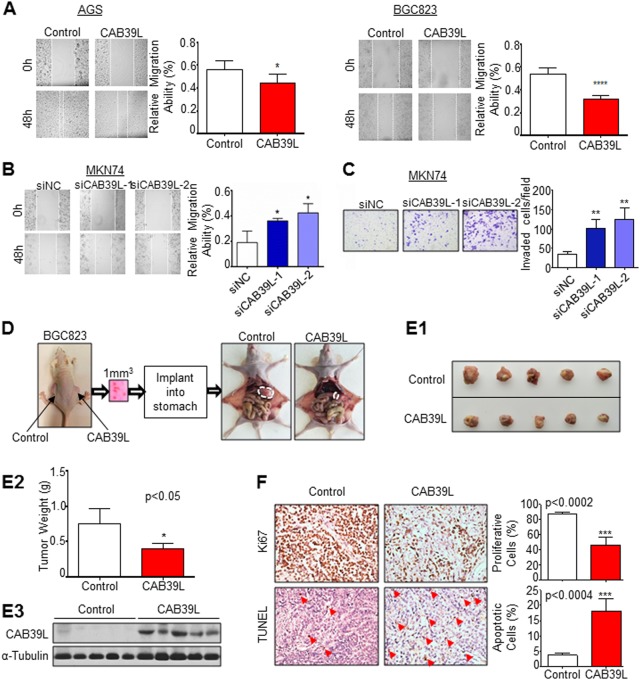
CAB39L suppressed cell migration/invasion in vitro and tumorigenicity in vivo. **a** CAB39L ectopic expression inhibited cell migration as determined by wound healing assay in AGS (*P* < 0.05) and BGC823 (*P* < 0.0001) cells. **b** Knockdown of CAB39L in MKN74 cells promoted cell migration (*P* < 0.05). **c** CAB39L Knockdown in MKN74 cells enhanced cell invasion (*P* < 0.01). **d** Establishment of an orthotopic xenograft model in mice stomach using BGC823 cells. **e1** CAB39L overexpression inhibited growth of orthotopic BGS823 xenograft in stomach. **e2** CAB39L reduced tumor weight (*P* < 0.05). **e3** Western blot confirmed CAB39L overexpression. **f** Ki-67 and TUNEL staining revealed a significant decrease in cell proliferation (*P* < 0.001) and induction of apoptosis (*P* < 0.001), respectively. (*P < 0.05; **P < 0.01; ***P < 0.001; ****P < 0.001)

As a member of the calcium binding protein family, CAB39L is implicated to function in cytoskeletal rearrangement and cell motility [24]. We hence determined the effect of CAB39L on cell migration and invasion by wound healing and Matrigel invasion assays, respectively. CAB39L overexpression suppressed cell migration as indicated by the reduced wound closure efficiency in both AGS and BGC823 cells (Fig. 3a, AGS: *P* < 0.05; BGC823: *P* < 0.0001). Conversely, silencing of CAB39L in MKN74 cells significantly increased both cell migration and invasion (Fig. 3b, c). Taken together, these results indicated that CAB39L suppressed GC cell phenotypes related to metastasis.

### CAB39L inhibits the growth of orthotopic GC xenografts

Based on our in vitro results, we next tested the effect of CAB39L on tumor growth in vivo. To this end, we utilized an orthotopic GC xenograft model using BGC823 cells. Orthotopic transplantation is able to better reproduce the organ microenvironment in which tumor grows [25]. In this orthotopic model, we first subcutaneously implanted BGC823 cells stably expressing CAB39L or empty vector into the dorsal flanks of nude mice. Developed tumors were harvested, trimmed to 1 mm^3^ cubes and then implanted into the stomach lining to generate orthotopic xenografts (Fig. 3d). In keeping with in vitro results, BGC823 xenografts with ectopic CAB39L expression demonstrated a significant reduction in both tumor size and weight (Fig. 3e1–2), with western blot confirming the ectopic expression of CAB39L (Fig. 3e3). Ki-67 and TUNEL staining revealed reduced cell proliferation (*P* < 0.0002) but increased apoptosis (*P* < 0.0004) in BGC823 xenografts overexpressing CAB39L (Fig. 3f). These data implied a tumor suppressive role of CAB39L in vivo.

### CAB39L activates AMPK pathway

**Fig. 4:**
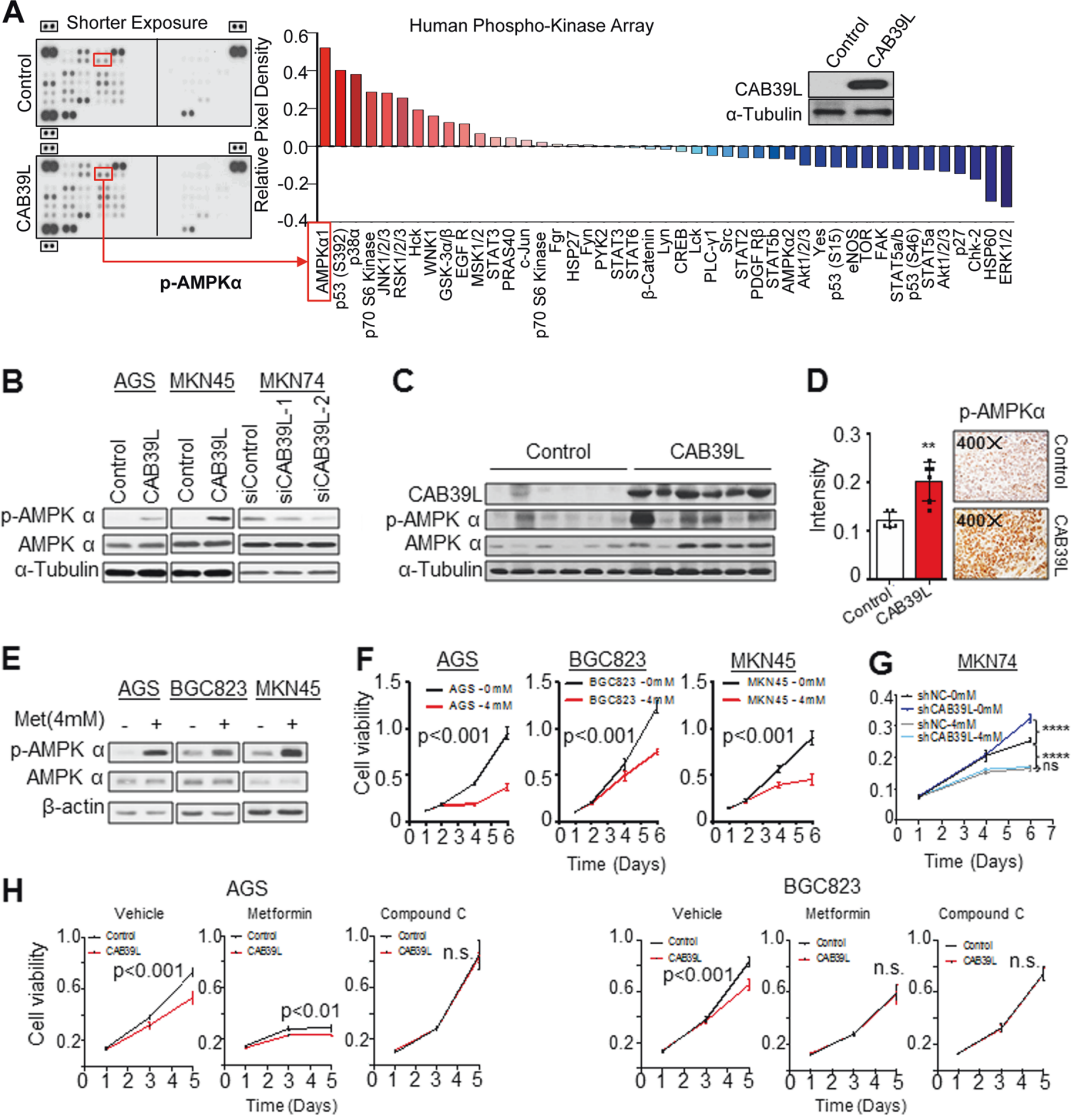
CAB39L positively regulated AMPK pathway. **a** Human phospho-kinase array analysis of BGC823 cells overexpressing CAB39L or empty vector. p-AMPKα was the top up-regulated kinase upon CAB39L overexpression. **b** AMPKα activation (phosphorylation) by CAB39L was validated by western blot analysis in two CAB39L overexpression cell lines (AGS and MKN45 cells). Conversely, CAB39L knockdown in MKN74 cells with siRNAs suppressed AMPKα phosphorylation. **c** Orthotopic resected tumors from mice revealed increased expression of p-AMPKα in CAB39L overexpression group compared to control group. **d** Immunohistochemistry for p-AMPKα in orthotopic xenografts showed increased p-AMPKα in CAB39L overexpression group compared to control group. **e** Metformin (4 mM) treatment in AGS, BGC823 and MKN45 cells activated AMPK pathway. **f** Metformin (4 mM) treatment suppressed the cell viability of AGS, BGC823 and MKN45 cells (*P* < 0.001). **g** Effect of metformin treatment on the viability of MKN74 cells expressing shControl and shCAB39L. **h** Metformin (4 mM) and Compound C (0.5 μM) demonstrated the tumor-suppressive role of CAB39L through AMPK pathway. (*P < 0.05; **P < 0.01; ***P < 0.001; ****P < 0.001)

Calcium binding protein 39 protein family have been reported to function as scaffold proteins involved in the activation of kinases [26]. To profile the kinase activation in GC cells, we performed the Phospho-Kinase Arrays to decipher the phosphorylation profiles of 43 cancer-related kinases. Using BGC823 cells overexpressing CAB39L, we identified AMPKα as the top kinase activated by CAB39L (Fig. 4a). To confirm the data of the antibody array, we performed western blot of AGS and MKN45 cells with ectopic CAB39L expression; and MKN74 cells with CAB39L knockdown (Fig. 4b). CAB39L overexpression induced the phosphorylation of p-AMPKα; whereas AMPK phosphorylation was inhibited with CAB39L knockdown. AMPKα is the catalytic subunit of AMPK protein complex (α, β, γ) and its phosphorylation is rate-limiting for AMPK activity [27]. Moreover, orthotopic BGC823 xenografts overexpressing CAB39L also demonstrated increased phosphorylation of p-AMPKα, consistent with in vitro data (Fig. 4c). In addition, we performed p-AMPKα immunohistochemistical staining in the orthotopic xenografts, which confirmed that CAB39L overexpression increased AMPKα phosphorylation in vivo (Fig. 4d). To ask if AMPK activation might mediate a tumor suppressive effect in GC, we treated three GC cell lines with Metformin, an AMPK activator commonly used for the treatment of type-2 diabetes. Metformin treatment markedly induced phosphorylation of AMPKα (Fig. 4e). Moreover, it consistently decreased cell viability among the GC cell lines assessed (Fig. 4f), suggesting that AMPK exerts a tumor suppressive effect in GC. To this end, we treated MKN74 cells expressing shNC or shCAB39L with metformin and examined cell viability. As expected, stable knockdown of CAB39L promoted cell proliferation in MKN74 cells (Fig. 4g, *P* < 0.0001), whereas metformin treatment suppressed the growth of MKN74-shCAB39L cells to a greater extent as compared to MKN74-shNC cells, thereby abrogating the tumor promoting effect of CAB39L loss (Fig. 4g, *P* < 0.0001). This result suggests that re-activation of AMPK using metformin can selectively target GC with transcriptional silencing of CAB39L. Consistently, control AGS and BGC823 cells (with silencing of CAB39L) were more sensitive to metformin compared to their CAB39L-overexpressing counterparts (Fig. 4h). Conversely, compound C (AMPK inhibitor) treatment rescued the growth inhibitory effect of CAB39L overexpression (Fig. 4h). These results underlie the important role of AMPK activation in the tumor suppressive function of CAB39L.

### CAB39L directly interacts with LKB1-STRAD, upstream of AMPK

**Fig. 5:**
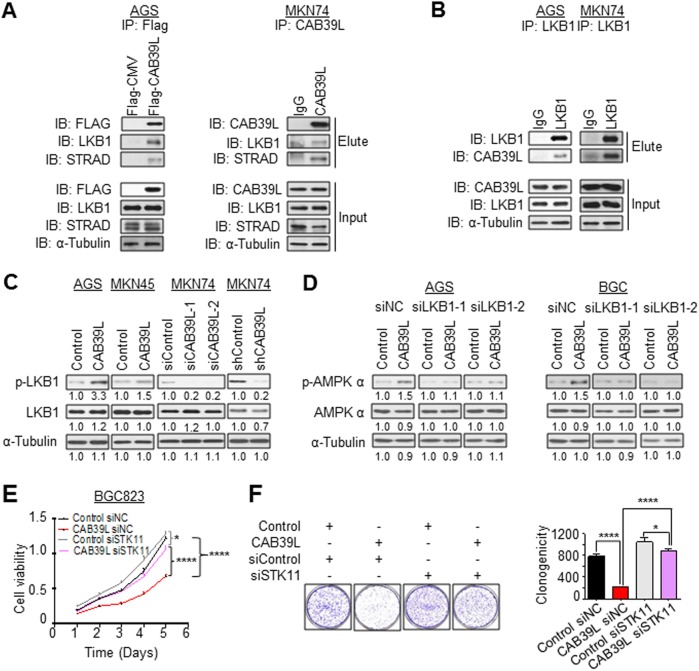
CAB39L interacted with LKB1/STRAD leading to the activation of LKB1. **a** Co-immunoprecipitation (Co-IP) of CAB39L with anti-Flag and anti-CAB39L in AGS-CAB39L and MKN74 cells, respectively, identified LKB1 and STRAD as the binding partners of CAB39L. **b** Reciprocal Co-IP confirmed the protein interaction between LKB1 and CAB39L in both AGS and MKN74 cell lines. **c** CAB39L overexpression in AGS and MKN45 cells induced p-LKB1; while knockdown of CAB39L in MKN74 cells reduced LKB1 phosphorylation. **d** LKB1 (STK11) silencing abolished the effect of CAB39L overexpression on AMPKα phosphorylation activation. **e**, **f** LKB1 silencing abolished tumor suppressive effect of CAB39L in BGC832 cells, as determined by **e** cell viability and **f** colony formation assays, suggesting that the effect of CAB39L was dependent on LKB1. (*P < 0.05; **P < 0.01; ***P < 0.001; ****P < 0.001)

CAB39, a paralog of CAB39L, has been reported to interact with LKB1 and STE20-Related Kinase Adaptor (STRAD), leading to activation of LKB1, a bona fide tumor suppressor and an upstream kinase that phosphorylates AMPK [28]. To evaluate whether CAB39L might function in a similar manner by binding to LKB1 and STRAD, we therefore performed co-IP in two GC cell lines: AGS with ectopic expression of Flag-tagged CAB39L and MKN74 with high endogenous CAB39L expression to validate protein-protein interactions. Concordantly, CAB39L pulldown with anti-Flag (AGS cells) or anti-CAB39L (MKN74 cells) successfully identified LKB1 and STARD as the binding partners for CAB39L (Fig. 5a). Reciprocal co-IP using anti-LKB1 in both cell lines also found CAB39L as an interacting protein (Fig. 5b). CAB39L expression promoted phosphorylation of LKB1 in AGS and MKN45 cells; whereas its knockdown in MKN74 cells produced an opposite effect (Fig. 5c), suggesting that CAB39L functions similarly to its paralog in interacting with LKB1-STRAD complex and driving LKB1 activation. Hence, CAB39L likely activates AMPK pathway through its interaction with LKB1.

### Tumor suppressive effect of CAB39L is dependent on LKB1-AMPK pathway

Given that CAB39L strongly activated LKB1-AMPK pathway in GC, we next sought to determine whether the tumor suppressive effect of CAB39L is dependent on LKB1 and AMPK activation. Firstly, we tested phosphorylation level of AMPKα in AGS and BGC823 cell lines with siLKB1 treatment. Results showed that LKB1 knockdown abolished activation of AMPKα by CAB39L in both cell lines (Fig. 5d). Furthermore, we performed siRNA-mediated LKB1 knockdown in BGC823 cells with or without CAB39L overexpression, and examined cell growth using cell growth curve and colony formation assays. As shown in Fig. 5e, f, knockdown of LKB1 largely abolished growth inhibitory effect of CAB39L in MTT and colony formation assays, suggesting that tumor suppressive function of CAB39L is dependent on LKB1. Given that the silencing of CAB39L in GC would inhibit LKB1-AMPK pathway to promote tumorigenesis, we hypothesized that the pharmacological modulation of AMPK activity may be a promising strategy to overcome CAB39L loss. The tumor suppressive effect of CAB39L is therefore dependent on downstream LKB1-AMPK pathway.

### CAB39L elicits an anti-Warburg effect by inducing PGC1α-mediated oxidative phosphorylation

**Fig. 6:**
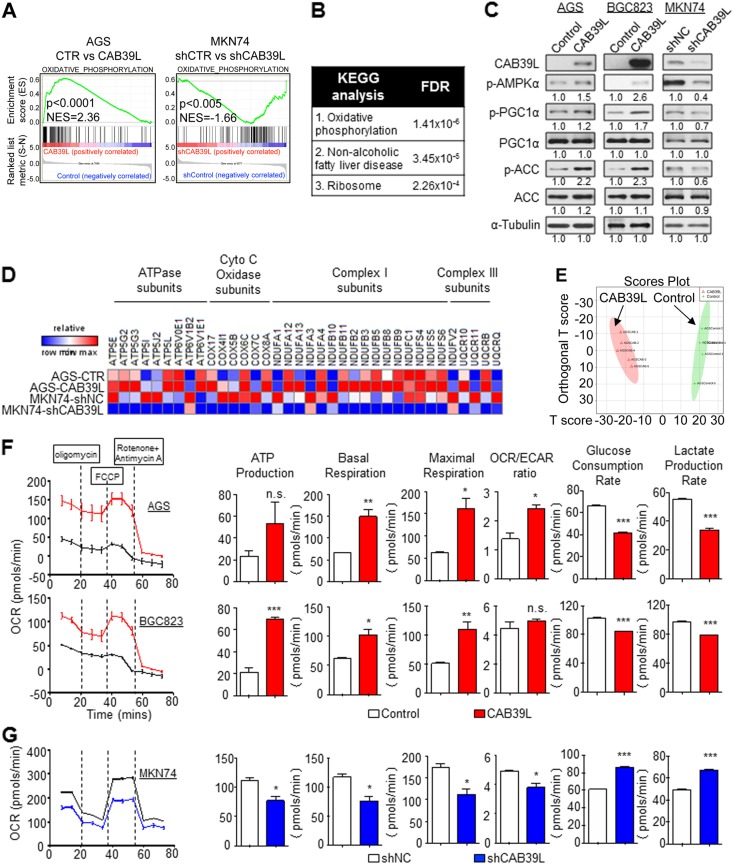
CAB39L exerted an anti-Warburg effect in gastric cancer cells. **a** RNA-seq analysis and Gene Set Enrichment Analysis (GSEA) analysis demonstrated that the transcriptome profile of AGS-CAB39L cells was positively enriched for oxidative phosphorylation compared to control AGS cells; while CAB39L silencing in MKN74 cells was negatively correlated with oxidative phosphorylation. **b** Top three KEGG pathways associated with CAB39L in gastric cancer cell lines. **c** Overexpression of CAB39L induced PGC1α phosphorylation, whilst CAB39L knockdown produced an opposite effect. **d** CAB39L positively regulated the expression of multiple enzymes involved in oxidative phosphorylation, as evidenced by gain-of-function and loss-of-function experiments. **e** Non-targeted metabolomics profiling of AGS cells stably expressing empty vector or CAB39L. Ortho-partial least square-discriminant analysis (OPLS-DA) of the metabolite profile showed a clear separation of AGS-control and AGS-CAB39L. **f** Effect of ectopic CAB39L expression on oxygen consumption rate (OCR) and extracellular acidification rate (ECAR). AGS and BGC823 cells with over-expression of CAB39L showed significant increase in basal OCR, maximal OCR and the OCR-to-ECAR ratio and decreased lactate production rate. **g** In MKN74 cells, knockdown of CAB39L decreased ATP production, basal/maximal respiration, OCR-to-EACR ratio and increased lactate production rate. (*P < 0.05; **P < 0.01; ***P < 0.001; ****P < 0.001)

To elucidate the downstream molecular mechanisms of CAB39L, we compared the transcriptome of AGS cells with ectopic CAB39L expression and MKN74 cells with shCAB39L expression by RNA sequencing. Gene set enrichment analysis (GSEA) of transcriptome profiles of AGS cells expressing empty vector or CAB39L unveiled that oxidative phosphorylation was the top pathway enriched in CAB39L overexpressing cells (NES = 2.36, *P* < 0.0001) (Fig. 6a). On the contrary, the expression profiles of MKN74 cells with knockdown of CAB39L were negatively associated with oxidative phosphorylation (NES = −1.66; *P* < 0.005) (Fig. 6a). In addition, top three pathways enriched in CAB39L overexpressing AGS cells, including oxidative phosphorylation, non-alcoholic fatty liver disease and ribosome, were all associated with mitochondrial biogenesis (Fig. 6b). PGC1α is a master regulator of mitochondria biogenesis [29] and a downstream substrate of AMPK. Western blot showed that overexpression of CAB39L induced the phosphorylation of PGC1α in AGS and BGC cell lines; whereas knockdown of CAB39L reduced p-PGC1α expression (Fig. 6c), suggesting that CAB39L may promote mitochondria biogenesis via activation of PGC1α. Indeed, genes associated with ATPase (ATP5E, ATP5G2, ATP5G3, ATP5I, ATP5J2, ATP5L, ATP6V0E1, ATP6V1B2, and *ATP6V1E1*), Cytochrome C Oxidase (*COX17, COX4I1, COX5B, COX6C, COX7C*, and *COX8A*), Complex I (*NDUFA1, NDUFA12, NDUFA13, NDUFA3, NDUFA4, NDUFB10, NDUFB11, NDUFB2, NDUFB3, NDUFB5, NDUFB8, NDUFB9, NDUFC1, NDUFS4, NDUFS5, NDUFS6, and NDUFV2*), and Complex II (*UQCR10, UQCR11, UQCRB, UQCRQ*) were commonly up-regulated in AGS cells expressing CAB39L as compared to control cells, whilst being simultaneously down-regulated by shCAB39L in MKN74 cells (Fig. 6d).

In light of the impact of CAB39L on metabolic pathways, we performed non-targeted metabolomics (UPLC-MS) profiling of AGS cells stably expressing empty vector and CAB39L. Ortho-Partial Least Square-Discriminant Analysis (OPLS-DA) of metabolite profiles demonstrated the clear separation of AGS-Control and AGS-CAB39L cells (Fig. 6e), suggesting that CAB39L significantly modified the metabolome of AGS cells. Altered metabolites were mainly associated with fatty acid oxidation, indicative of increased fatty catabolism in mitochondria in order to support increased oxidative phosphorylation (Figure S2). This was in agreement with increased phosphorylation of acetyl-CoA carboxylase [30] (Fig. 6c) leading to inactivation of ACC and promotion of fatty acid oxidation. To test the degree to which metabolism of GC cells was altered by CAB39L-induced oxidative phosphorylation, we measured key indexes of mitochondrial metabolism, including oxygen consumption rate (OCR) and extracellular acidification rate, using XF24 Seahorse Analyzer. In AGS and BGC823 cells, CAB39L overexpression significantly induced basal and maximal OCR, which was accompanied by increased ATP production (Fig. 6f). OCR-to-EACR ratio was increased in AGS by CAB39L, suggesting that metabolism was shifted from glycolysis towards an oxidative phenotype (Fig. 6f). Additionally, we estimated glycolysis flux by measuring glucose uptake and lactate secretion. Consistent with the reversal of the Warberg effect, CAB39L overexpression suppressed glucose consumption and lacetate fermentation in AGS and BGC823 cells (Fig. 6f, *P* *<* *0*.0001). Conversely, shCAB39L in MKN74 cells triggered transition towards a glycolytic phenotype, as reflected by decreased basal/maximal OCR, ATP production and increased glucose consumption and lactate secrection (Fig. 6g, *P* *<* 0.0001). These results indicated that CAB39L elicited an anti-Warburg effect by promoting oxidative phosphorylation and suppressing the glycolytic phenotype, and implied that CAB39L functions as a metabolic checkpoint silenced by promoter hypermethylation in GC.

### CAB39L hypermethylation correlate with poor outcomes in GC patients

**Fig. 7:**
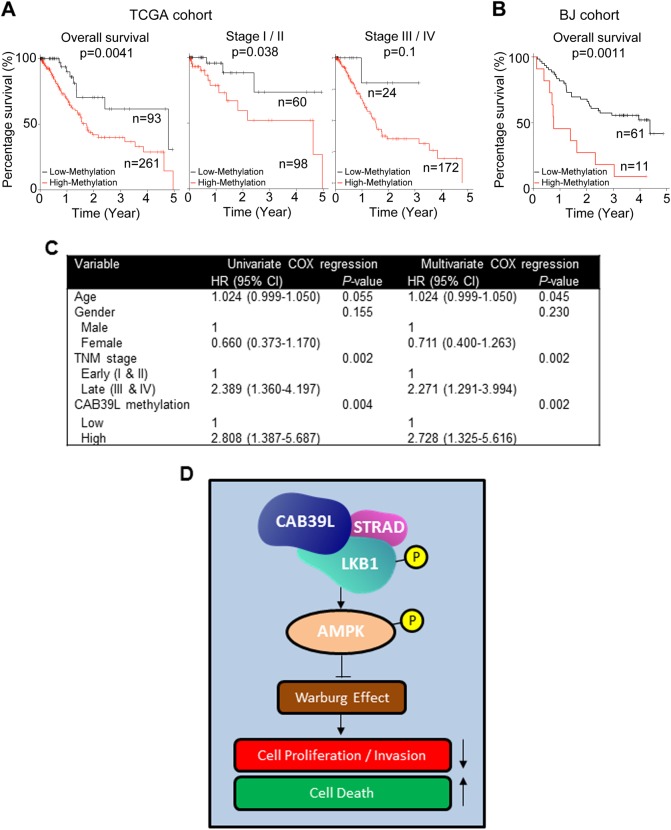
Clinical implication and molecular mechanism of CAB39L in gastric cancer. **a** Analysis of TCGA DNA methylation data (27 K/450 K) showed that high CAB39L promoter methylation predicted poor survival in gastric cancer, especially for early stage (I/II) gastric cancer. **b** CAB39L promoter hypermethylation was associated with poor survival in gastric cancer (BJ cohort). **c** Univariate and multivariate Cox regression analysis of potential survival predictor for patients with gastric cancer in the TCGA cohort. **d** Molecular mechanism of action of CAB39L in gastric cancer. CAB39L interacts with LKB1 and promotes LKB1 phosphorylation, which in turn, activates AMPK and PGC1α. CAB39L-induced LKB1-AMPK-PGC1α axis produced an anti-Warburg effect, involving activation of oxidative phosphorylation concomitant with suppression of glycolysis. The re-establishment of an oxidative phenotype suppressed cell proliferation/invasion and induced cell death in gastric cancer cells

To evaluate the association between CAB39L promoter methylation and outcomes of GC patients, we determined the promoter methylation of CAB39L in 72 primary GC patients using BGS. With the best cut-off value determined by ROC curve analysis, Kaplan-Meier curves demonstrated that GC patients with high promoter methylation (*n* = 11) had poorer survival compared to those with low promoter methylation (*n* = 61) (Fig. 7b). Consistent with our cohort, analyses of TCGA GC cohort (*n* = 354, cg15207619) also revealed that CAB39L promoter hypermethylation was associated with poor survival (Fig. 7a). Stratification of TCGA GC cohort into early (I/II) and late (III/IV) stages showed that CAB39L promoter hypermethylation was correlated with poor survival in early stage, but not late stage GC patients (Fig. 7a). Moreover, multivariate COX proportional hazards regression analysis of the TCGA cohort revealed that CAB39L high promoter methylation was an independent prognostic factor that predicts shorter survival (*P* = 0.002; hazard ratio, 2.728; 95% CI, 1.325–5.616) (Fig. 7c). Hence, CAB39L promoter methylation could serve as an independent prognostic biomarker for GC patients.

## Discussion

In this study, we demonstrated that CAB39L is readily expressed in normal gastric tissues, but silenced in most GC cell lines and primary GC tissues, which is concomitant with CAB39L promoter hypermethylation. Silencing of CAB39L is mediated by promoter DNA methylation, as demethylation treatment restored CAB39L expression in GC cell lines. The frequent silencing of CAB39L in GC suggests that it might function as a tumor suppressor in GC.

Using in vitro and in vivo models of GC, we demonstrated the tumor suppressive function of CAB39L. Consistent with our hypothesis, ectopic expression of CAB39L in three GC cell lines (AGS, BGC823 and MKN45) significantly inhibited cell proliferation and colony formation; whilst the knockdown of CAB39L in MKN74 cells, which express high endogenous CAB39L, promoted cell proliferation. The anti-proliferative effect of CAB39L was exerted via induction of apoptosis and blockade of cell cycle progression. Apoptosis mainly involved the extrinsic apoptosis pathway, as indicated by sequential activation of caspase-8, -7 and -3. CAB39L suppressed cell cycle progression by activating G1-checkpoints p21 and p27. Using an orthotopic xenograft model that mimics the native tumor microenvironment in stomach, we validated the tumor suppressive effect of CAB39L in vivo, and decreased cell proliferation in CAB39L-expressing orthotopic xenografts was confirmed by Ki-67 staining. CAB39L also restrained the metastatic capacity of GC cells by suppressing cell migration and invasion. Hence, the down-regulation of CAB39L in GC may contribute to tumor development and progression.

Interplay between epigenetics and tumor metabolism is increasingly recognized as an important mechanism contributing to tumorigenesis. Accumulating evidence indicate that deregulated metabolic phenotypes in tumors are driving forces of tumorigenesis in several cancers, such as lung and breast cancers [31–33]. However, to date the molecular mechanisms contributing to deregulated metabolism in GC remain poorly defined. Our work identified that the aberrant silencing of CAB39L in GC by promoter hypermethylation represent an epigenetic mechanism contributing to metabolic dysregulation and GC development. Our mechanistic investigations have pinpointed the LKB1-AMPK pathway as a major metabolic pathway functioning downstream of CAB39L. CAB39, and its paralog CAB39L, are scaffolding proteins that participate in protein complexes involving STE20-related kinases and pseudo-kinases, leading to their activation [26, 34]. Co-immunoprecipitation revealed direct interaction between CAB39L and LKB1/STRAD (a STE20-like pseudokinase), and CAB39L overexpression could drive activation of LKB1 in GC cells. LKB1, a serine-threonine kinase, is a *bona fide* tumor suppressor mutationally inactivated in many sporadic cancers [35] and Peutz-Jeghers syndrome, a rare disease associated high risk of gastrointestinal cancers [36]. However, LKB1 is infrequently mutated (2%) in GC. Hence, CAB39L promoter hypermethylation and transcriptional silencing in GC might constitute an alternative pathway to inactivate LKB1.

LKB1 transduces its signals via direct phosphorylation of AMPK at Thr172 located in the activation loop of α-subunit, leading to subsequent AMPK activation [27, 34, 35, 37]. Consistent with LKB1 activation, CAB39L promoted phosphorylation and activation of AMPKα in GC cells and in orthotopic xenografts in nude mice. AMPK is a pivotal metabolic sensor that functions to maintain cellular energy homeostasis and couple energy signals to growth control [35, 38]. Energy stress as manifested by increased AMP/ATP ratio activates AMPK, which in turn positively regulates energy production by promoting fatty acid oxidation and oxidative phosphorylation in mitochondria via PGC1α;[39] while shutting down energy-intensive anabolic processes as well as cell proliferation [40]. AMPK also suppresses aerobic glycolysis, as it is an inefficient way of energy production [41]. The constellation of events mediated by AMPK generates an anti-Warburg effect and represses tumorigenesis [39, 41]. In line with its effect on AMPK, overexpression of CAB39L in GC cell lines triggered a repertoire of cellular responses consistent with AMPK activation, including up-regulation of p-PGC1α, oxidative phosphorylation (exemplified by increased OCR) and fatty acid oxidation, thus culminating in an anti-Warburg effect to suppress tumorigenesis (Fig. 6). Conversely, silencing of CAB39L promoted a glycolytic phenotype and cell growth. Our data indicates that CAB39L constitutes a metabolic checkpoint by up-regulating LKB1-AMPK-PGC1α cascade, thereby limiting the metabolic transition to a Warburg phenotype in GC cells.

CAB39L-silenced GC cells exhibited an increased sensitivity to metformin. Metformin has shown promising anticancer activities in multiple human cancers [42–44]. Considering that CAB39L is commonly silenced in gastric cancer patients, metformin may serve as a potential adjuvant therapy for GC treatment. The clinical significance of CAB39L in GC was further evidenced by the association of CAB39L promoter hypermethylation with GC patient outcomes. High CAB39L promoter methylation was correlated with poor patient survival in two independent GC cohorts (Fig. 7a–c). CAB39L methylation may thus serve as a novel biomarker for the prognosis of GC patients.

In summary, we identified CAB39L as a novel tumor suppressor that is frequently silenced by promoter hypermethylation in GC. Mechanistically, CAB39L exerts an anti-Warburg effect through a LKB1-AMPK-PGC1α cascade (Fig. 7d), which leading to the suppression of GC. CAB39L serves as a potential prognostic biomarker for GC patients.

## Materials and methods

### GC cell lines

AGS and NCI-N87 cells were purchased from the American Type Culture Collection (ATCC, Manassas, VA). MKN1, MKN74, MKN45, SNU638, SNU668, and SNU719 cells were purchased from the Korean Cell Line Bank (Seoul, Korea). MKN7 cells were ordered from RIKEN Cell Bank. BGC823, HGC27, MGC803, SGC7901, TMK1 and normal gastric cell line GES1 were acquired from the Cell Bank of Chinese Academy of Sciences (Shanghai, China). All cell lines have been authenticated and confirmed negative for mycoplasma contamination by providers.

### Human samples

Paired primary GC and adjacent normal gastric tissues were collected at the Prince of Wales Hospital and Peking University Cancer Hospital and Institute. Samples were collected after surgical resection and stored at -80 °C. Two normal gastric mucosa biopsies were obtained during gastroscopy. Histological examination of all GC and adjacent normal tissues was performed by an experienced pathologist at the Prince of Wales Hospital. All patients gave informed consent and the study protocol was approved by the Clinical Research Ethics Committee of the Chinese University of Hong Kong and Peking University.

### Cell culture

All cells were routinely cultured in DMEM (Gibco BRL, Rockville, MA) supplemented with 10% fetal bovine serum and 1% Anti-Anti. Transient transfection was performed with Lipofectamine 2000, following the manufacturer’s protocol. Stable CAB39L overexpression or knockdown cell lines were established using lentivirus infection and selection with 2 ng/μl puromycin.

### Primers, plasmid, siRNAs and shRNA

Bisulfite genomic sequencing primers: Forward: 5′-GATTAATTAATGGGTTTTTGGTTG-3′; Reverse: 5′-AAAAATACTTCACCATTTACTAACCTTC-3′. Methylation-specific PCR primers: M forward: GTATTTTAGATGAAGTAGTTTTTATC; U forward: GTATTTTAGATGAAGTAGTTTTTATT; M reverse: TAAAAACTCTTTTCTCTACCACG; U reverse: TAAAAACTCTTTTCTCTACCACA. qPCR primers: Forward 5′-GGCCTGCTAGTGACACTGATA-3′; Reverse 5′-TACTCCACAGTAGGACTCCGA-3′. CAB39L plasmid: pLvx-PGK-Puro-3 × Flag-hCAB39L. siNC and shNC are obtained from Ribobio Co. (Guangzhou, China) and OriGene Co. (Rockville, USA), respectively.

siCAB39L-1 target sequence: CCTACTGTGGAGTATATTA.

siCAB39L-2 target sequence: GTTGGTAGCAGACTTCTTA.

shCAB39L target sequence: GTGGAGTATATTAGTGCTCAT.

siSTK11-1 target sequence: GAAGAAGGAAATTCAACTA.

siSTK11-2 target sequence: GCACGCGGCTTGTTGACTT.

### Colony formation and cell viability assays

For colony formation assay, cells (0.5–1.0 × 10^3^/well) were seeded into 24-well plates. Cells were stained with 0.5% crystal violet after 14–20 days. Cell viability was performed using the 3-(4,5-Dimethylthiazol-2-yl)-2,5-diphenyltetrazolium bromide (MTT) assay. Cells (1–2 × 10^3^/well) were plated into 96-well plates to generate cell growth curves.

### Apoptosis and cell cycle analysis

Cell apoptosis was accessed by using Annexin-phycoerythrin/7-aminoactinomycin D (Annexin V-PE/7-AAD) staining kit (BD Biosciences, San Jose, CA). For cell cycle analysis, cells were serum-starved overnight, following by stimulation with complete medium for 6-8 h. Cells were fixed with 75% ethanol, stained using propidium iodide (PI) (BD Biosciences) and analyzed by flow cytometry.

### Wound healing assay

Confluent cells in 6-well plates were scratched with sterilized tips, washed with PBS and cultured in serum-free medium. Images were captured at 0, 24, 48 and 72 h. Wound closure (%) was calculated and analyzed.

### Cell invasion assay

Cell invasion was evaluated using the Matrigel Invasion Chamber (BD Biosciences). Cells (1–3 × 10^5^) were seeded into the upper chamber in serum-free DMEM medium. DMEM with 10% FBS as a chemoattractant was added into the lower chamber. After 24-48 h, cells that have successfully invaded through the upper chamber membrane were stained with 0.5% crystal violet and analyzed.

### Orthotopic gastric cancer mouse model

BGC823 cells (1 × 10^7^ cells in 100 μL PBS) with or without ectopic CAB39L expression were injected subcutaneously into C57BL/6 male nude mice (3–4 weeks) to form xenografts after 13 days. Subcutaneous tumors were cut into 1.0 mm^3^ cubes and then implanted into the gastric wall of nude mice (6 mice/group) randomly. The number of animals were determined based on our experience, where 6 mice will be sufficient to demonstrate ~50% difference in tumor growth. Mice were harvested after three weeks and the tumor weights were measured. The investigators were blinded to group allocation during the experimental procedures. All animal procedures were approved by the Animal Ethics Committee of the Chinese University of Hong Kong.

### Phospho-kinase array

Total proteins (400 μg) were incubated overnight with the Human Phospho-Kinase Array (R&D Systems, MN), which were spotted with antibodies for 43 human kinases and 2 related proteins. Signal detection was performed according to manufacturer’s instructions. The signal intensity of each spot was determined by ImageJ (NIH, USA).

### Co-immunoprecipitation

Cells stably expressing CAB39L-FLAG or empty vector were lysed in ice-cold lysis buffer (50 mmol/L Tris-Cl, 150 mmol/L NaCl, 1% NP-40, 0.5% sodium deoxycholate, and 1% SDS, pH = 7.4) supplemented with and Protease and Phosphatase inhibitors. The total proteins (1–5 mg) were immunoprecipitated using 2 μg of anti-Flag (F1804, Sigma-Aldrich), anti-CAB39L (sc-100390, Santa Cruz Biotechnology) or anti -LKB1 (sc-32245, Santa Cruz Biotechnology) antibodies, together with Protein A/G Magnetic Beads (Merck Millipore). After washing with washing buffer for three times, immunoprecipitated proteins were eluted by heating in loading buffer at 100 °C for 5–8 mins.

### Metabolomic Analysis

AGS cells (5 × 10^7^ cells) were harvested in 80% methanol. Global metabolomics was acquired from Ultimate 3000 rapid separation liquid chromatography (RSLC) coupled with Q Exactive Focus MS (Thermo Scientific, USA) as previously described [23].

### RNA sequencing

5 × 10^7^ cells were seeded in complete medium overnight. RNA was then extracted in TRIzol™ Reagent (Thermo Fisher Scientific). RNA sequencing was performed using AGS stably expressing empty vector or CAB39L, and MKN74 cells stably expressing shControl or shCAB39L. Duplicate samples were submitted to Groken Bioscience Limited (Hong Kong) for RNA sequencing analysis (6GB of data per sample). Gene set enrichment analysis (GSEA) was performed to identify the molecular pathways correlated with CAB39L in GC using GSEA, version 2.0. GSEA was performed using the KEGG geneset (c2.cp.kegg.v6.1.symbols.gmt). One thousand permutations were performed and gene sets with false discovery rate ≤0.01 and nominal *p*-value ≤0.01 were considered significantly enriched.

### Agilent seahorse XF cell mito stress test

Cells (1.5–2 × 10^4^/well) were seeded to 96-well Seahorse XF Cell Culture Microplate overnight. All procedures in the following day are conducted according to the Agilent Seahorse XF Cell Mito Stress Test protocol with FCCP concentration at 0.5 μM.

### Statistical analysis

All results were presented as mean ± S.D. All in vitro experiments were performed in triplicates and were repeated at least twice independently. Student’s *t*-test or Mann–Whitney *U* test was performed to compare the means between two groups. Two-way analysis of variance was used for comparing growth curves. The effect of CAB39L promoter methylation on patient survival was analyzed by the Kaplan-Meier survival curve and log-rank test. Variance similar between the groups were statistically compared. *P* < 0.05 was considered as statistically significant.

## Electronic supplementary material

Supplementary File

Supplementary Figure 1

Supplementary Figure 2

Supplementary Figure 3

## References

[1] Camargo MC, Kim WH, Chiaravalli AM, Kim KM, Corvalan AH, Matsuo K (2014). Improved survival of gastric cancer with tumour Epstein-Barr virus positivity: an international pooled analysis. Gut.

[2] Van Cutsem E, Sagaert X, Topal B, Haustermans K, Prenen H (2016). Gastric cancer. Lancet.

[3] Macdonald JS, Smalley SR, Benedetti J, Hundahl SA, Estes NC, Stemmermann GN (2001). Chemoradiotherapy after surgery compared with surgery alone for adenocarcinoma of the stomach or gastroesophageal junction. New Engl J Med.

[4] Das M (2017). Neoadjuvant chemotherapy: survival benefit in gastric cancer. Lancet Oncol.

[5] Jacome AA, Coutinho AK, Lima EM, Andrade AC, dos Santos JS (2016). Personalized medicine in gastric cancer: Where are we and where are we going?. World J Gastroenterol.

[6] Selaru FM, David S, Meltzer SJ, Hamilton JP (2009). Epigenetic events in gastrointestinal cancer. Am J Gastroenterol.

[7] Calcagno DQ, Gigek CO, Chen ES, Burbano RR, Smith MDC (2013). DNA and histone methylation in gastric carcinogenesis. World J Gastroenterol.

[8] Wang K, Liang Q, Li X, Tsoi H, Zhang J, Wang H (2016). MDGA2 is a novel tumour suppressor cooperating with DMAP1 in gastric cancer and is associated with disease outcome. Gut.

[9] Yu J, Cheng YY, Tao Q, Cheung KF, Lam CN, Geng H (2009). Methylation of protocadherin 10, a novel tumor suppressor, is associated with poor prognosis in patients with gastric cancer. Gastroenterology.

[10] Xu L, Li X, Chu ES, Zhao G, Go MY, Tao Q (2012). Epigenetic inactivation of BCL6B, a novel functional tumour suppressor for gastric cancer, is associated with poor survival. Gut.

[11] Qian Y, Wong CC, Xu J, Chen H, Zhang Y, Kang W (2017). Sodium channel subunit SCNN1B suppresses gastric cancer growth and metastasis via GRP78 degradation. Cancer Res.

[12] Wong CC, Qian Y, Yu J (2017). Interplay between epigenetics and metabolism in oncogenesis: mechanisms and therapeutic approaches. Oncogene.

[13] Gupta V, Gopinath P, Iqbal MA, Mazurek S, Wellen KE, Bamezai RNK (2014). Interplay between epigenetics & cancer metabolism. Curr Pharm Des.

[14] Liberti MV, Locasale JW (2016). The Warburg effect: how does it benefit cancer cells?. Trends Biochem Sci.

[15] Vander Heiden MG, Cantley LC, Thompson CB (2009). Understanding the Warburg effect: the metabolic requirements of cell proliferation. Science.

[16] Wellen KE, Hatzivassiliou G, Sachdeva UM, Bui TV, Cross JR, Thompson CB (2009). ATP-citrate lyase links cellular metabolism to histone acetylation. Science.

[17] Xiao MT, Yang H, Xu W, Ma SH, Lin HP, Zhu HG (2012). Inhibition of alpha-KG-dependent histone and DNA demethylases by fumarate and succinate that are accumulated in mutations of FH and SDH tumor suppressors. Gene Dev.

[18] Xu W, Yang H, Liu Y, Yang Y, Wang P, Kim SH (2011). Oncometabolite 2-hydroxyglutarate is a competitive inhibitor of alpha-ketoglutarate-dependent dioxygenases. Cancer Cell.

[19] Liu X, Wang X, Zhang J, Lam EKY, Shin VY, Cheng ASL (2010). Warburg effect revisited: an epigenetic link between glycolysis and gastric carcinogenesis. Oncogene.

[20] Dong CF, Yuan TT, Wu YD, Wang YF, Fan TWM, Miriyala S (2013). Loss of FBP1 by snail-mediated repression provides metabolic advantages in basal-like breast cancer. Cancer Cell.

[21] Garrido N, Martinez-Conejero JA, Jauregui J, Horcajadas JA, Simon C, Remohi J (2009). Microarray analysis in sperm from fertile and infertile men without basic sperm analysis abnormalities reveals a significantly different transcriptome. Fertil Steril.

[22] Rahmioglu N, Macgregor S, Drong AW, Hedman AK, Harris HR, Randall JC (2015). Genome-wide enrichment analysis between endometriosis and obesity-related traits reveals novel susceptibility loci. Hum Mol Genet.

[23] Li XN, Chung ACK, Li SF, Wu LL, Xu JY, Yu J (2017). LC-MS-based metabolomics revealed SLC25A22 as an essential regulator of aspartate-derived amino acids and polyamines in KRAS-mutant colorectal cancer. Oncotarget.

[24] Khanna C, Wan XL, Bose S, Cassaday R, Olomu O, Mendoza A (2004). The membrane-cytoskeleton linker ezrin is necessary for osteosarcoma metastasis. Nat Med.

[25] Richmond A, Su Y (2008). Mouse xenograft models vs GEM models for human cancer therapeutics. Dis Model Mech.

[26] Filippi BM, de los Heros P, Mehellou Y, Navratilova I, Gourlay R, Deak M (2011). MO25 is a master regulator of SPAK/OSR1 and MST3/MST4/YSK1 protein kinases. Embo J.

[27] Mihaylova MM, Shaw RJ (2011). The AMPK signalling pathway coordinates cell growth, autophagy and metabolism. Nat Cell Biol.

[28] Zeqiraj E, Filippi BM, Deak M, Alessi DR, van Aalten DMF (2009). Structure of the LKB1-STRAD-MO25 complex reveals an allosteric mechanism of kinase activation. Science.

[29] LeBleu VS, O’Connell JT, Gonzalez Herrera KN, Wikman H, Pantel K, Haigis MC (2014). PGC-1alpha mediates mitochondrial biogenesis and oxidative phosphorylation in cancer cells to promote metastasis. Nat Cell Biol.

[30] Mariotti S, Barravecchia I, Vindigni C, Pucci A, Balsamo M, Libro R (2016). MICAL2 is a novel human cancer gene controlling mesenchymal to epithelial transition involved in cancer growth and invasion. Oncotarget.

[31] William WN, Kim JS, Liu DD, Solis L, Behrens C, Lee JJ (2012). The impact of phosphorylated AMP-activated protein kinase expression on lung cancer survival. Ann Oncol.

[32] Morales DR, Morris AD (2015). Metformin in cancer treatment and prevention. Annu Rev Med.

[33] Nagalingam A, Arbiser JL, Bonner MY, Saxena NK, Sharma D (2012). Honokiol activates AMP-activated protein kinase in breast cancer cells via an LKB1-dependent pathway and inhibits breast carcinogenesis. Breast Cancer Res.

[34] Boudeau J, Baas AF, Deak M, Morrice NA, Kieloch A, Schutkowski M (2003). MO25alpha/beta interact with STRADalpha/beta enhancing their ability to bind, activate and localize LKB1 in the cytoplasm. EMBO J.

[35] Shackelford DB, Shaw RJ (2009). The LKB1-AMPK pathway: metabolism and growth control in tumour suppression. Nat Rev Cancer.

[36] Hearle N, Schumacher V, Menko FH, Olschwang S, Boardman LA, Gille JJ (2006). Frequency and spectrum of cancers in the Peutz-Jeghers syndrome. Clin Cancer Res.

[37] Faubert B, Vincent EE, Poffenberger MC, Jones RG (2015). The AMP-activated protein kinase (AMPK) and cancer: many faces of a metabolic regulator. Cancer Lett.

[38] Hardie DG, Ross FA, Hawley SA (2012). AMPK: a nutrient and energy sensor that maintains energy homeostasis. Nat Rev Mol Cell Biol.

[39] Xing F, Luan YZ, Cai J, Wu SH, Mai JL, Gu JY (2017). The anti-warburg effect elicited by the cAMP-PGC1 alpha pathway drives differentiation of glioblastoma cells into astrocytes. Cell Rep.

[40] Motoshima H, Goldstein BJ, Igata M, Araki E (2006). AMPK and cell proliferation - AMPK as a therapeutic target for atherosclerosis and cancer. J Physiol.

[41] Faubert B, Boily G, Izreig S, Griss T, Samborska B, Dong ZF (2013). AMPK Is a negative regulator of the warburg effect and suppresses tumor growth in vivo. Cell Metab.

[42] Whitburn J, Edwards CM, Sooriakumaran P (2017). Metformin and prostate cancer: a new role for an old drug. Curr Urol Rep.

[43] Meng FQ, Song L, Wang WY (2017). Metformin improves overall survival of colorectal cancer patients with diabetes: a meta-analysis. J Diabetes Res.

[44] Camacho L, Dasgupta A, Jiralerspong S (2015). Metformin in breast cancer - an evolving mystery. Breast Cancer Res.

