# Supplementary material for: CAB39L elicited an anti-Warburg effect via a LKB1-AMPK-PGC1α axis to inhibit gastric tumorigenesis
Source: Oncogene. 2018 Jul 27;37(50):6383–98. doi: 10.1038/s41388-018-0402-1 (PMC6296350; doi:10.1038/s41388-018-0402-1)
Supplement: Supplementary file 1 — Supplementary File [file 41388_2018_402_MOESM1_ESM.docx]

**Supplementary Figure 1.** (A) Infinium Human Methylation 450 BeadChip analysis revealed that CpGs within the CAB39L locus are hypermethylated in GC cell lines as compared to the normal gastric epithelial cell line GES1 and normal gastric tissues. (B) Analysis of the TCGA gastric cancer 27K methylation and RNA sequencing datasets identified CAB39L as one of the top outlier (0.5%) genes silenced via promoter methylation

**Supplementary Figure 2.** CAB39L Promoter DNA methylation was inversely correlated with mRNA expression in the TCGA gastric cancer cohort.

**Supplementary Figure 3.** Tumor suppressive effect of CAB39L in cell proliferation. (A) CAB39L inhibits cell proliferation in low-concentration of Glucose and Glutamine which mimic physiological condition. (B) Re-expression of CAB39L in CAB39L-knockdwon cell line MKN74 restores its tumor suppressive effect in cell proliferation.

**Supplementary Table - Antibody List**

| Antibodies | Supplier | Species | Type | Dilution | Reference |
| --- | --- | --- | --- | --- | --- |
| CAB39L | Santa Cruz | Mouse | Monoclonal | 1/1000 | sc-100390 |
| β-actin | Santa Cruz | Mouse | Monoclonal | 1/1000 | sc-47778 |
| α-Tubulin | Santa Cruz | Mouse | Monoclonal | 1/1000 | sc-8035 |
| Cleaved-Caspase-3 | Cell signalling | Rabbit | Polyclonal | 1/1000 | 9661 |
| Cleaved-Caspase-7 | Cell signalling | Rabbit | Polyclonal | 1/1000 | 9491 |
| Cleaved-Caspase-8 | Cell signalling | Mouse | Monoclonal | 1/1000 | 9496s |
| Caspase-3 | Cell signalling | Rabbit | Monoclonal | 1/1000 | 9665 |
| Caspase-7 | Cell signalling | Rabbit | Polyclonal | 1/1000 | 9492 |
| Cleaved-PARP | Cell signaling | Rabbit | Monoclonal | 1/1000 | 5625 |
| PARP | Cell signaling | Rabbit | Monoclonal | 1/1000 | 9532 |
| P21 | Santa Cruz | Mouse | Monoclonal | 1/1000 | sc-6246 |
| P27 | Cell signalling | Rabbit | Monoclonal | 1/1000 | 3686 |
| AMPK α | Cell signalling | Rabbit | Monoclonal | 1/1000 | 9957 |
| p-AMPK α (Western Blot) | Cell signalling | Rabbit | Monoclonal | 1/1000 | 9957 |
| p-AMPK α (IHC) | Thermo Fisher | Rabbit | Polyclonal | 1/1000 | PA5-36764 |
| Flag-tag | Sigma-Aldrich | Mouse | Monoclonal | 1/1000 | F1804 |
| LKB1 | Santa Cruz | Mouse | Monoclonal | 1/1000 | sc-374334 |
| p-LKB1 | Cell signalling | Rabbit | Monoclonal | 1/1000 | 3482 |
| STRAD | Santa Cruz | Mouse | Monoclonal | 1/1000 | sc-293230 |
| PGC1 α | Cell signalling | Rabbit | Monoclonal | 1/1000 | 2178s |
| p-PGC1 α | R&D Systems | Rabbit | Polyclonal | 1/1000 | AF6650 |
| ACC | Cell signalling | Rabbit | Monoclonal | 1/1000 | 9957 |
| p-ACC | Cell signalling | Rabbit | Monoclonal | 1/1000 | 9957 |
