# Supplementary figures and images for: CAB39L elicited an anti-Warburg effect via a LKB1-AMPK-PGC1α axis to inhibit gastric tumorigenesis
Source: Oncogene. 2018 Jul 27;37(50):6383–98. doi: 10.1038/s41388-018-0402-1 (PMC6296350; doi:10.1038/s41388-018-0402-1)

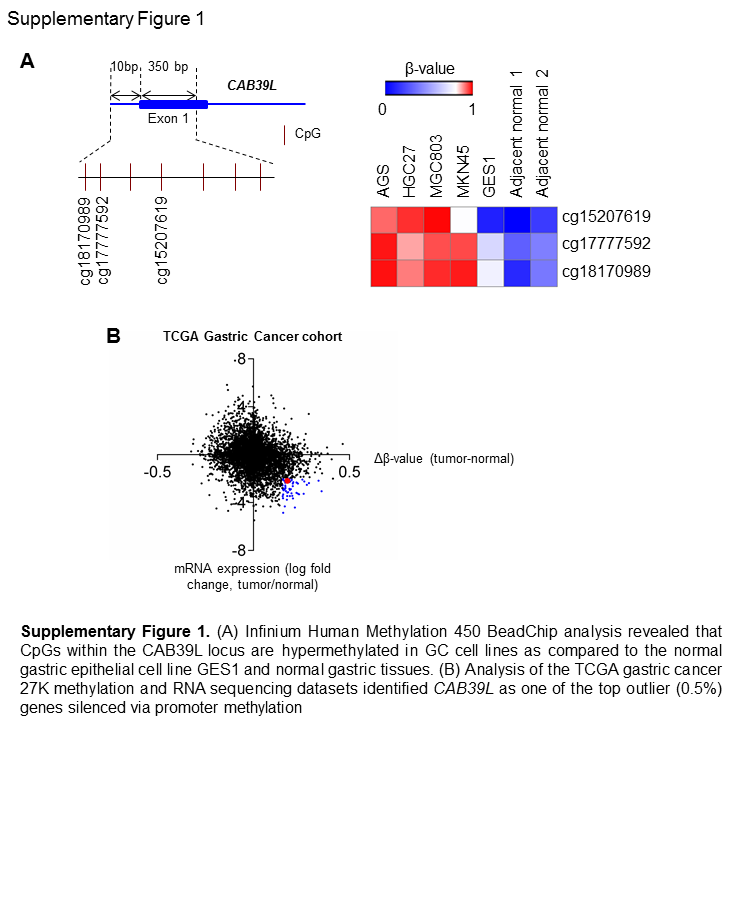

Supplement: Supplementary file 2 — Supplementary Figure 1 [file 41388_2018_402_MOESM2_ESM.tif]

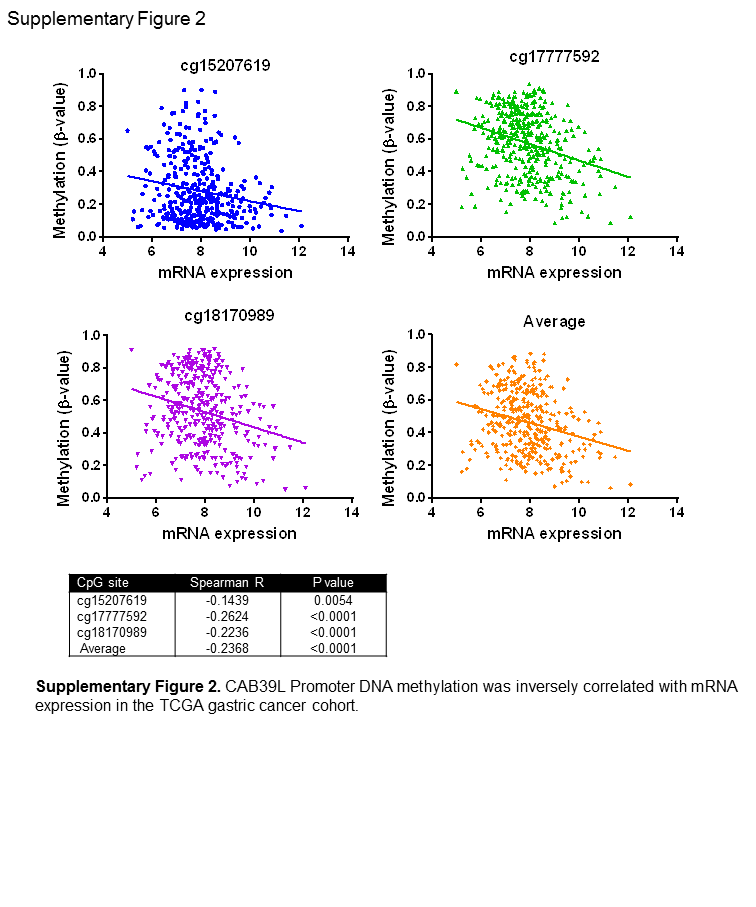

Supplement: Supplementary file 3 — Supplementary Figure 2 [file 41388_2018_402_MOESM3_ESM.tif]

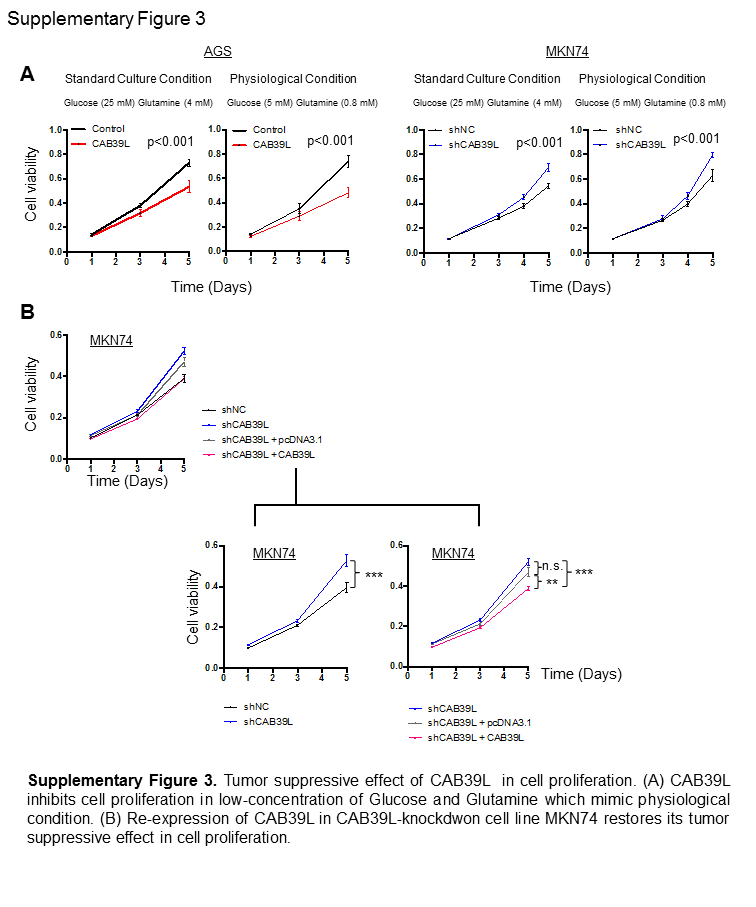

Supplement: Supplementary file 4 — Supplementary Figure 3 [file 41388_2018_402_MOESM4_ESM.tif]
